# Identification of potential human targets for epigallocatechin gallate through a novel protein binding site screening approach

**DOI:** 10.1007/s00894-025-06410-y

**Published:** 2025-06-13

**Authors:** Jernej Hirci, Sandra Škufca, Tanja Kunej, Dušanka Janežič, Janez Konc

**Affiliations:** 1 Theory Department, National Institute of Chemistry, Hajdrihova 19, SI-1001 Ljubljana, Slovenia; 2 Department of Animal Science, Biotechnical Faculty, University of Ljubljana, Groblje 3, 1230, Domzale, Slovenia; 3 Faculty of Mathematics, Natural Sciences and Information Technologies, University of Primorska, Glagoljaška ulica 8, SI-6000 Koper, Slovenia; 4 Faculty of Pharmacy, University of Ljubljana, Aškerčeva 7, SI-1000 Ljubljana, Slovenia

**Keywords:** Epigallocatechin-3-gallate, ProBiS-Dock algorithm, ProBiS-Dock Database, Inverse molecular docking, Target protein prediction

## Abstract

**Context:**

Epigallocatechin-3-gallate (EGCG), a compound found in green tea, is known for its anticancer properties, although its specific protein targets remain largely undefined. In this study, we identified EGCG targets across the human proteome using a novel protein binding site screening approach. Among the 20 most likely predicted targets, six proteins—KRAS, FXa, MMP1, PLA2G2A, Hb, and CDK2—had been experimentally validated in previous studies. Fourteen additional proteins, including five kinases, were newly predicted as potential targets, all of which are implicated in cancer development and may mediate EGCG’s anticancer effects. Enrichment analysis revealed KEGG pathways associated with cancer, with KRAS and PIM1 appearing as key nodes. These findings, which align with previous experimental research, offer new insights into the molecular mechanisms of EGCG and its potential role in modulating cancer-related pathways.

**Methods:**

An approach was devised to screen EGCG with 36,532 human protein binding sites using the ProBiS-Dock algorithm and the ProBiS-Dock database. Network and enrichment analyses with Cytoscape and StringApp identified protein interactions and KEGG pathways, revealing potential anticancer mechanisms of EGCG.

**Graphical Abstract:**

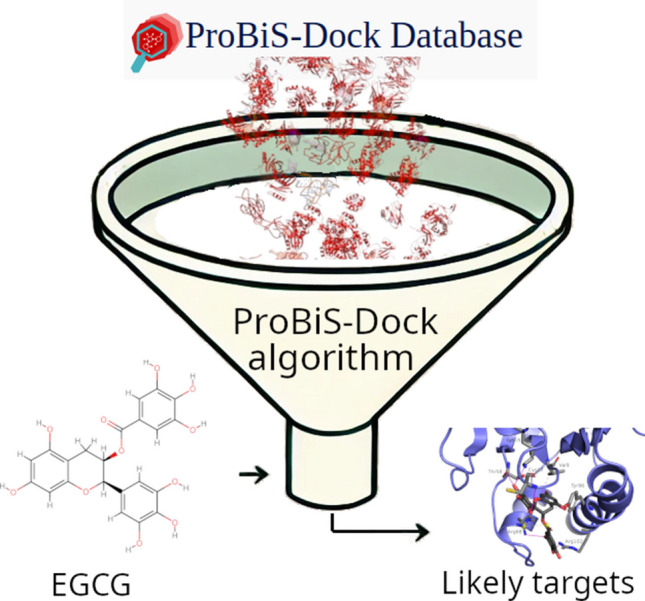

**Supplementary Information:**

The online version contains supplementary material available at 10.1007/s00894-025-06410-y.

## Introduction

**Fig. 1:**
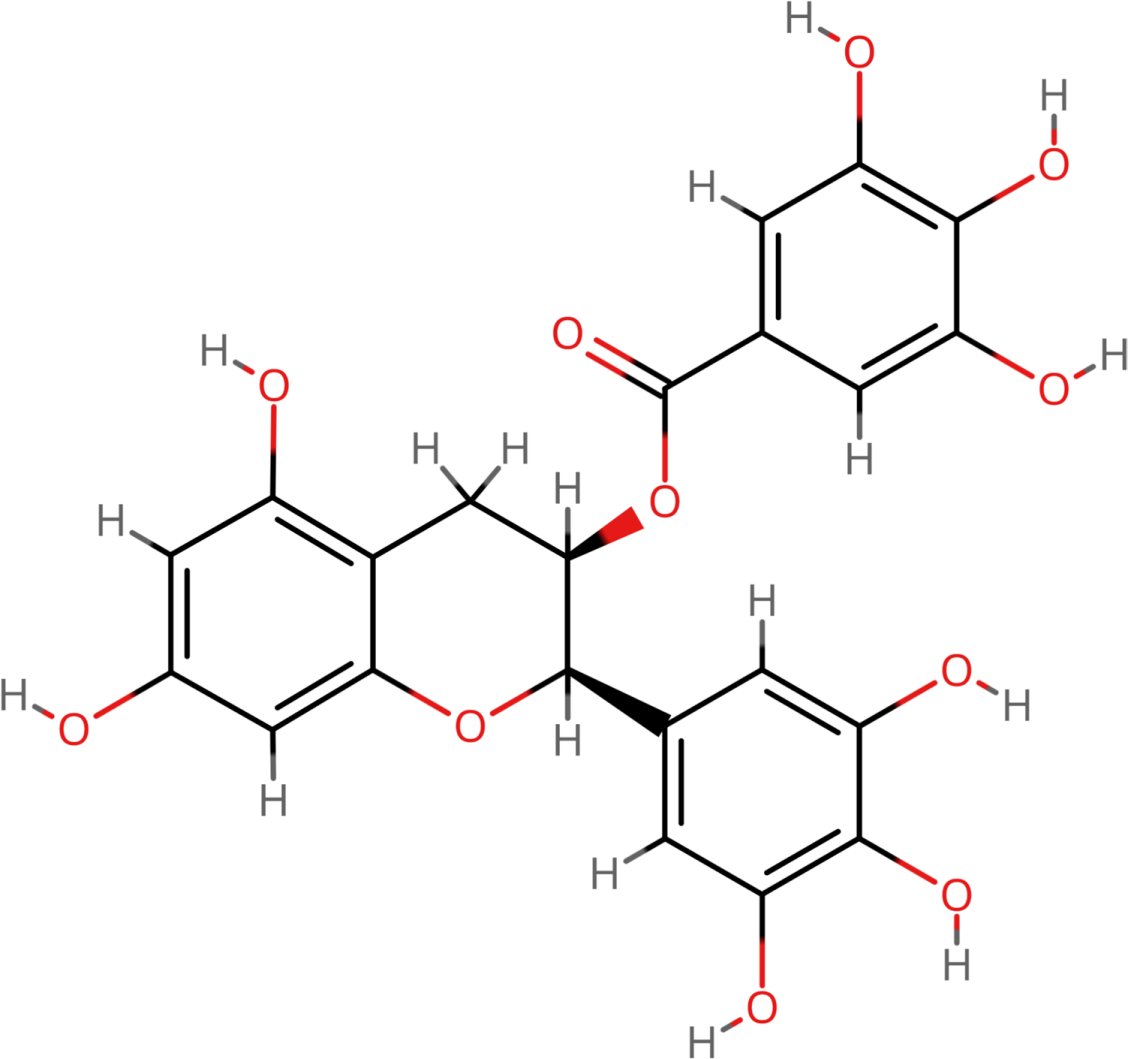
EGCG molecule

Green tea, derived from *Camellia sinensis*, is the second most consumed beverage in the world after water [1]. It is rich in polyphenolic catechins, which make up 30–40% of its dry weight [2]. Among these, (−)-epigallocatechin-3-gallate (EGCG) (Fig. 1), a flavone-3-ol phenolic compound, is particularly notable for its bioactivity and health benefits, including antioxidant properties, metal chelation, and antibacterial potential [3, 4]. Epidemiological studies link the consumption of green tea, and EGCG in particular, to a lower risk of chronic diseases such as cardiovascular disease, diabetes, and cancer [5]. A typical cup of green tea contains 50 to 100 mg of EGCG, which is considered the most effective anticancer agent among the green tea polyphenols [6]. However, the bioavailability of EGCG is limited due to its polarity and intestinal metabolism [7].

EGCG exerts its anticancer effects through modulation of several signaling pathways and enzymes, making it a promising candidate for cancer treatment. Animal studies have demonstrated that EGCG can inhibit tumor growth, reduce tumor size and invasiveness, suppress angiogenesis, and induce apoptosis in cancer cells, suggesting potential therapeutic benefits in humans [8]. One notable mechanism of EGCG’s anticancer action is its ability to arrest the cell cycle of cancer cells at the G1 phase [9]. Additionally, EGCG induces apoptosis through both extrinsic and intrinsic pathways [10, 11], inhibits anti-apoptotic protein expression, and promotes pro-apoptotic protein expression in cancer cells [12].

At the epigenetic level, EGCG regulates gene expression by inhibiting enzymes such as DNMT and HDAC [13]. It inhibits DNMT3B in cervical cancer cells, thereby reducing the methylation of tumor suppressor gene promoters [14]. EGCG also modulates various intracellular enzymes including kinases [11] and proteinases [15] and immune regulators [16]. Furthermore, it inhibits the catalytic component of telomerase, which is frequently overexpressed in cancer cells [17].

EGCG demonstrates anti-inflammatory effects by suppressing the expression of inflammatory cytokines, growth factors, and chemokines [18–20], which could potentially aid in cancer prevention. Moreover, EGCG inhibits the expression of hormone and growth factor receptors that are often overexpressed in cancer cells [19, 21].

Drugs often interact with numerous proteins outside of their intended target, known as off-targets, which can significantly affect their overall activity, efficacy, tolerability, and side effects [22]. The fact that EGCG affects a broad spectrum of molecular signaling pathways, as indicated, suggests that it may engage in multiple off-target interactions. Despite numerous studies highlighting these polypharmacological effects, our understanding of the exact molecular mechanisms by which EGCG exerts its effects in the various metabolic pathways is still limited. The identification of new EGCG targets or pathways through in silico approaches such as ours could therefore shed light on the putative anticancer effects of EGCG.

We have developed two innovative tools, the ProBiS-Dock docking algorithm (http://insilab.org/probisdock) [23] and the ProBiS-Dock database (http://probis-dock-database.insilab.org) [24], to enable identification of new target proteins for synthetic and natural small molecules. The ProBiS-Dock algorithm treats both the protein and the ligand as flexible entities and uses a novel docking approach in which the scoring function utilizes information from similar binding sites with known ligands. The ProBiS-Dock database includes the predicted binding sites for all human proteins in the Protein Data Bank (PDB) [25]. It includes over 1.4 million predicted binding sites, of which more than 36,000 are on human protein structures. Together, these tools provide a comprehensive resource for inverse molecular docking, enabling scanning of the human proteome to identify potential targets.

**Fig. 2:**
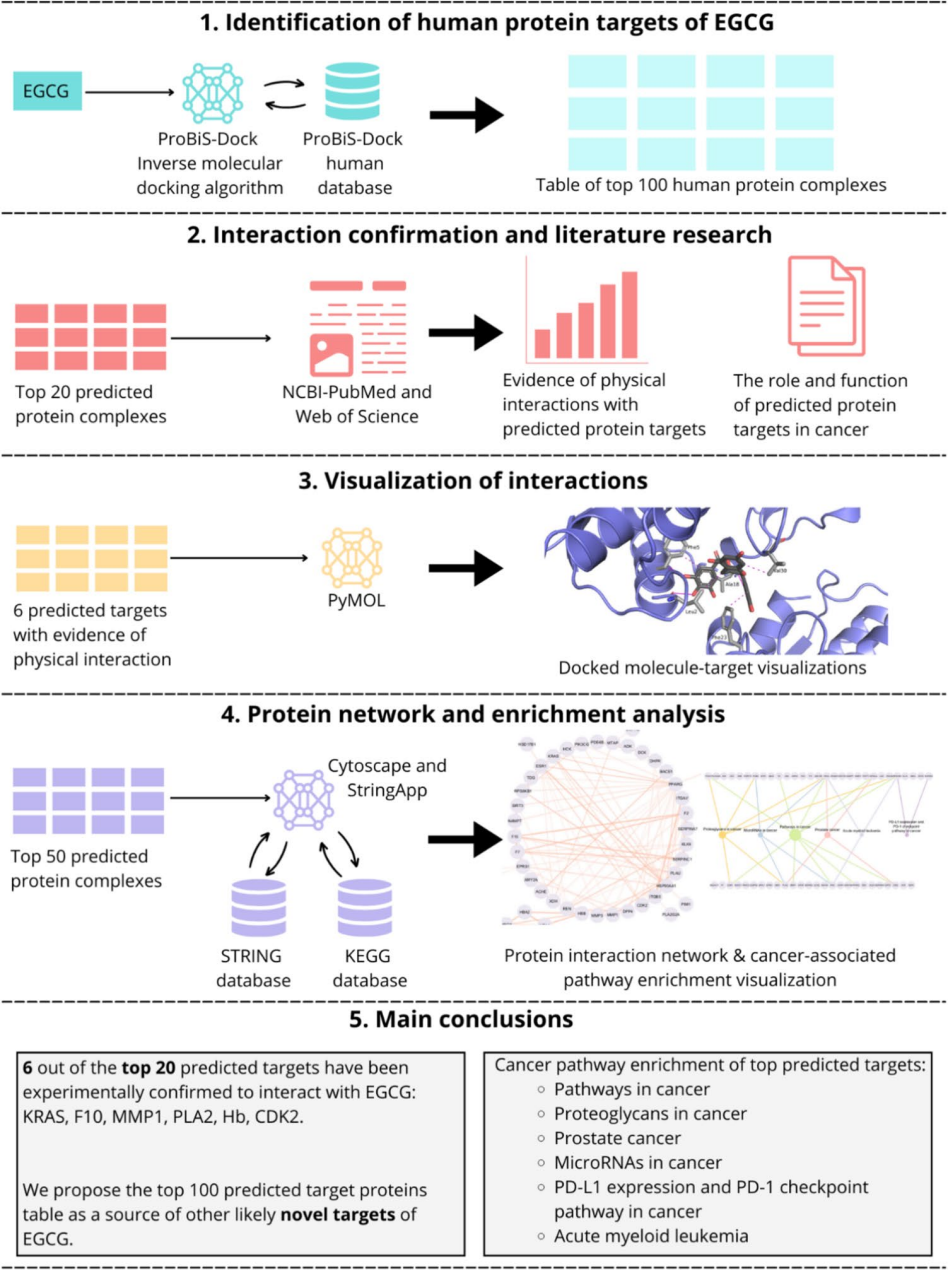
Overview of the study

In this study, we used these inverse docking tools to identify the most likely target proteins of EGCG (Fig. 2). We conducted a thorough search for experimental evidence of the physical interaction between EGCG and the predicted target proteins. For the top predicted target proteins, we provided detailed descriptions of their functions and roles, focusing particularly on their involvement in carcinogenesis. In addition, we performed molecular pathway enrichment analysis based on the best predictions to elucidate the potential signaling pathways affected by EGCG. Our study is an initial exploration into the interaction of EGCG with different proteins and aims to establish a list of potential human target proteins of EGCG to enable future research to investigate its anticancer effects.

## Materials and methods

### Identification of human protein targets of EGCG in the Protein Data Bank

Using the ProBiS-Dock algorithm [23] for molecular docking, we conducted a comprehensive search across the entire Protein Data Bank (PDB) [25] to identify human protein targets of EGCG. This algorithm has undergone extensive validation [23], and an earlier version of the algorithm [26] was used to predict new target proteins of different natural products [27, 28]. The algorithm treats small molecules and proteins as flexible entities, allowing for the simulation of conformational changes during the docking process. It features a novel scoring function, which incorporates specific ligand information from the PDB tailored to each protein structure, along with a general statistical scoring function. These components are integrated into a hybrid scoring function called ProBiS-Score that provides both structure-specific scoring and general scoring potentials applicable across all protein structures.

The ProBiS-Dock database [24] is a repository containing over 1.4 million small molecule ligand binding sites derived from all protein structures available in the PDB. Each binding site within this database is prepared for docking studies, encompassing cofactors, relevant metal ions, glycan molecules, and structural water molecules.

The biologically relevant ligands from similar proteins are transposed onto the corresponding query protein chains using rotational–translational matrices, provided the binding sites share sufficient structural similarity, as determined by ProBiS-assigned Z-scores. Ligand transfer occurs when Z-scores exceed 2.5 for compounds, cofactors, glycans, and water molecules, and 2.0 for metal ions.

These components are critical as they can interact with docked ligands. Importantly, the database facilitates the identification of binding sites spanning multiple protein chains that form complexes. These sites are often overlooked, as conventional methods typically recognize only the asymmetric unit of protein complexes, neglecting the entire multi-chain structure. Binding sites are ranked according to their druggability, that is, the suitability for drug targeting, which is assessed based on a novel druggability score that considers the complexity and number of predicted ligands for each site [24]. This is particularly important for allosteric binding sites where co-crystallized ligand-protein structures are scarce. In this work, we used the human subset of the ProBiS-Dock database, which comprises 440,723 binding sites [25]. We used the highest ranked binding site by druggability score among all predicted binding sites for each protein, resulting in 36,532 binding sites.

We ran ProBiS-Dock algorithm using the parameters max_possible_conf set to 20 and flex_radius set to 6.0, while keeping all other parameters at their default values.

### Protein targets selection based on docking scores

**Fig. 3:**
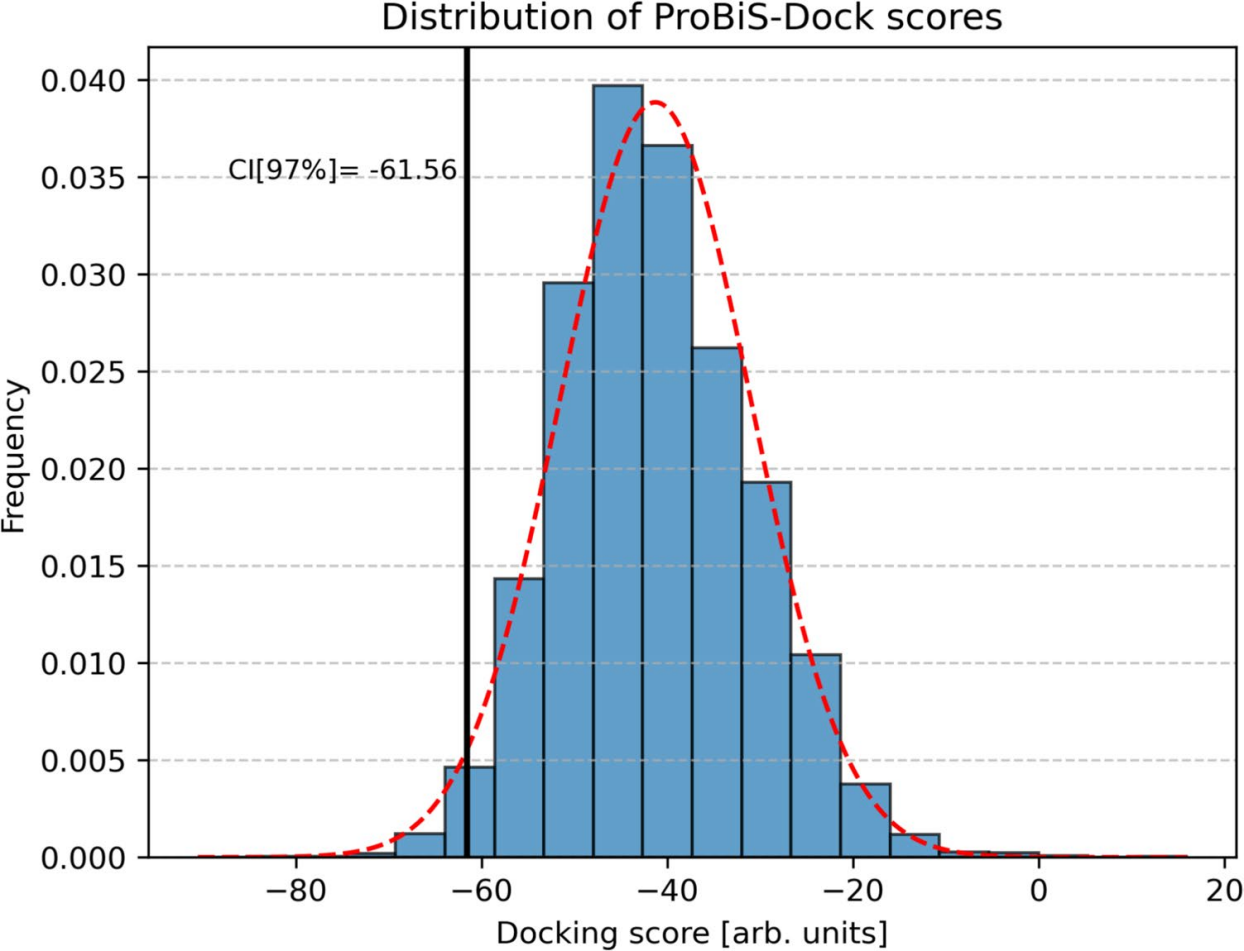
ProBiS-Dock score distribution (blue bars) with an overlaid normal distribution (dashed red line). The black vertical line represents the lower bound of the 97% confidence interval. The histogram is normalized to match the probability density function, ensuring accurate comparison with the normal distribution

The predicted conformations of EGCG in human protein binding sites were ranked based on their ProBiS-Dock scores. Assuming a normal distribution of these scores, the 97^th^ percentile confidence interval was determined to be (−61.56, −18.03) arbitrary units (Fig. 3). Proteins with ProBiS-Dock scores below this threshold (< −61.56 arb. units) were considered potential EGCG targets (see the full list in Supporting Table S1).

By setting the threshold at the 97^th^ percentile, we focused on only the top-scoring EGCG-protein complexes, selecting those most likely to represent true biological interactions. The identified 20 top-ranked protein targets were chosen for further manual investigation (Table 1), including a search for experimental evidence supporting their interaction with EGCG.

**Table 1:** Top 20 target proteins (including complexes composed of multiple protein chains) with the lowest ProBiS-Score for EGCG, predicted by the ProBiS-Dock algorithm

#	PDB ID, Chain ID	Protein name^*a*^	Gene symbol^*b*^	ProBiS-Score [23]	Interaction with EGCG described in the literature
1.	4LV6, B	GTPase KRas	KRAS	−90.530	^*C*^Yes [29]
2.	2D1 J, A	Coagulation factor X, heavy chain	F10	−83.500	^*C*^Yes [30]
3.	3ENM, AC	Dual specificity mitogen-activated protein kinase kinase 6	MAP2K6	−82.459	No
4.	4LVF, AB	Nicotinamide phosphoribosyltransferase	NAMPT	−82.124	No
5.	1IME, AB	Inositol monophosphatase	IMPA1	−81.353	No
6.	3 AYK, A	Collagenase	MMP1	−81.231	^*c*^Yes [31]
7.	3 WD9, A	CAMP-specific 3',5'-cyclic phosphodiesterase 4B	PDE4B	−79.799	No
8.	2 C0 T, A	Tyrosine-protein kinase HCK	HCK	−77.626	No
9.	1 J1 A, AB	Phospholipase A2	PLA2G2A	−74.696	^*C*^Yes [32]
10.	1ST4, AB	mRNA decapping enzyme	DCPS	−72.006	No
11.	1 CB0, A	5′-Deoxy-5′-methylthioadenosine phosphorylase	MTAP	−71.748	No
12.	4 J6I, A	Phosphatidylinositol 4,5-bisphosphate 3-kinase catalytic subunit gamma isoform	PIK3CG	−71.132	No
13.	3 W1B, A	DNA ligase 4	LIG4	−70.995	No
14.	3R01, A	Proto-oncogene serine/threonine-protein kinase pim-1	PIM1	−70.887	No
15.	1 FO3, A	Alpha 1,2-mannosidase	MAN1B	−70.779	No
16.	4 C7B, AB	NAD-dependent protein deacetylase sirtuin-3, mitochondrial	SIRT3	−70.313	No
17.	2I6 A, A	Adenosine kinase	ADK	−70.043	No
18.	2 JT5, A	Stromelysin-1	MMP3	−69.280	No
19.	4MQK, ABEF	Hemoglobin subunit alpha, subunit gamma-2	AE: *HBA1*, *HBA2* BF: *HBB*, *HBG2*	−69.226	^*D*^Yes [33]
20.	3R8M, A	Cyclin-dependent kinase 2	CDK2	−69.161	^*C*^Yes [34]

^*a*^Gene names were obtained from PDB (https://www.rcsb.org/)

^*b*^Gene symbols were obtained from UniProt (https://www.uniprot.org/)

^*c*^Interaction was confirmed by in vitro enzymatic activity assay

^*d*^Interaction was confirmed by in vitro Hb reduction

### Network and enrichment analysis

We performed network and enrichment analyses to determine the biological context and potential pathways associated with the predicted target proteins of EGCG through which EGCG exerts its anticancer effects. We used the top 50 predicted human proteins, and using Cytoscape 3.10.2 [35] with integrated with StringApp [36], we performed protein-protein interaction network analysis based on the STRING database [37]. In the StringApp, all parameters were set to their default values. For pathway enrichment analysis, we focused on KEGG (Kyoto Encyclopedia of Genes and Genomes) pathways [38] using StringApp within Cytoscape.

## Results and discussion

We used the inverse molecular docking tool ProBiS-Dock [23, 24] to predict human protein targets of EGCG. From the resulting list, we selected 95 the most likely protein targets with their docking scores in the 97 th percentile for further analysis. Among the top 20 predicted EGCG targets—an arbitrary yet manageable number that allowed manual evaluation—six have been experimentally confirmed to interact with EGCG (see Table 1, all targets are provided in Supporting Table S1). The remaining 14 target proteins are novel predictions and await experimental validation.

### Validation of protein targets based on literature evidence

For the top 20 EGCG target predictions, we conducted a literature search to find experimental evidence of physical interactions between EGCG and these targets. While many studies demonstrated effects of EGCG on the predicted targets, it was often not clear whether these effects were due to direct physical interactions or indirect mechanisms. Therefore, as a precaution, we confirmed only those interactions for which we found evidence in the literature that was obtained using a reliable experimental method, e.g., an in vitro enzyme activity assay. This led to the confirmation of six of the top 20 predicted targets as true physical interactions (see rows with “Yes” in the last column in Table 1). These are described in the following.

**Fig. 4:**
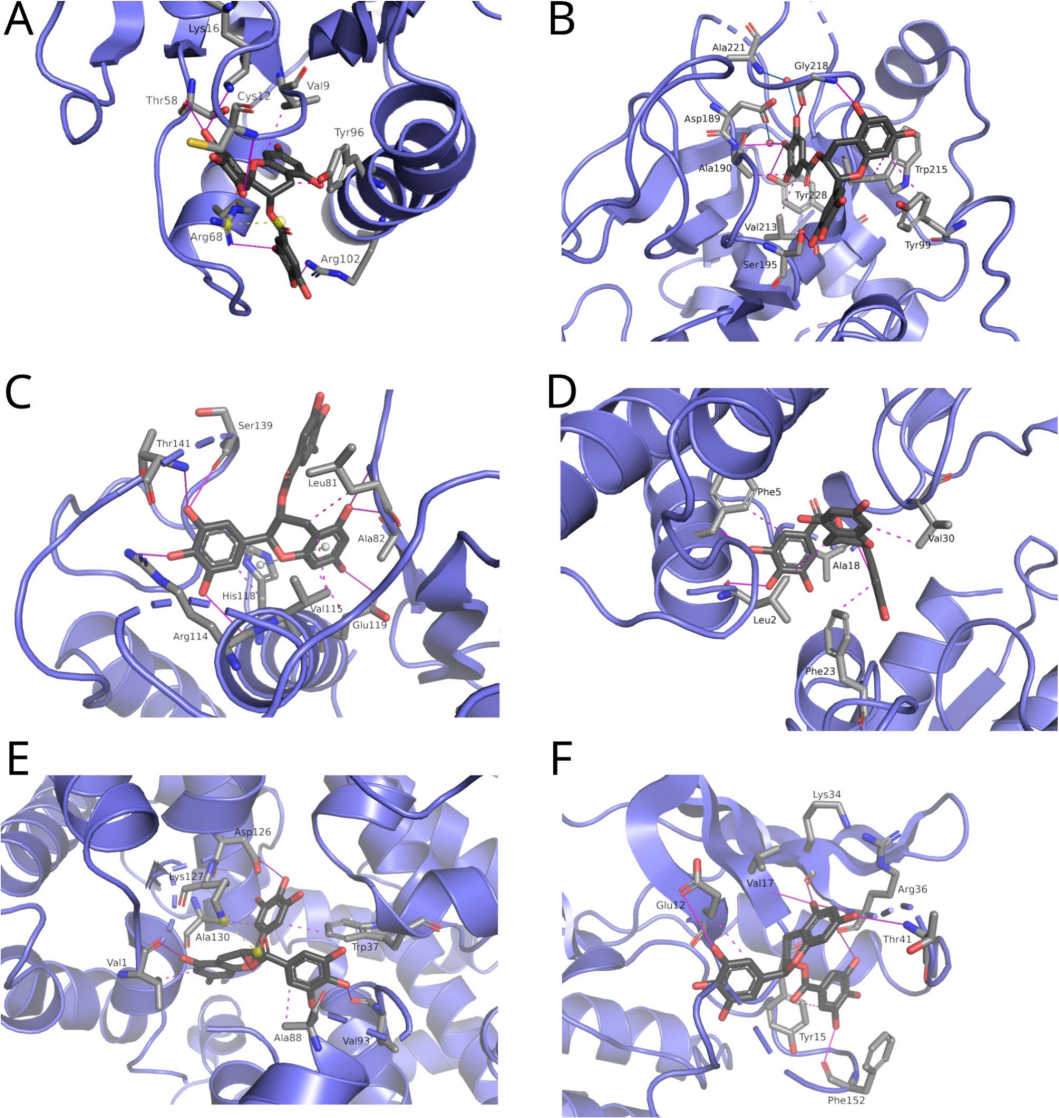
Predicted interactions between EGCG and **A** KRAS (PDB ID: 4LV6), **B** FXa (PDB ID: 2D1 J), **C** MMP1 (PDB ID: 3 AYK), **D** PLA2G2A (PDB ID: 1 J1 A), **E** Hb (PDB ID: 4MQK), and **F** CDK2 (PDB ID: 3R8M). Hydrogen bonds are continuous purple lines, hydrophobic interactions are purple dashed lines, salt bridges are yellow dashed lines, pi-pi interactions are dashed white lines, and water bridges are continuous blue lines

KRAS *GTPase* (KRAS), the most prevalent oncogenic driver gene in human cancers, emerged as the top predicted protein target of EGCG, with its docked conformation in KRAS shown in Fig. 4A. Experimental evidence demonstrates that EGCG indeed physically interacts with KRAS, leading to inhibition of its activity. Wang et al. [29] conducted a targeted study on cancer-associated protein targets of EGCG using an in vitro enzyme activity assay specific for KRAS. Their results showed that EGCG effectively inhibited KRAS activity by 53% at a concentration of 100 μM (see Fig. 8 in Ref. [29]). After incubation with the compound, enzymatic activity was measured colorimetrically at 450 nm using a microplate reader. Given the high prevalence and lethality of KRAS mutations, the ability of EGCG to inhibit KRAS activity confirms EGCG as a potential therapeutic option for cancers caused by KRAS mutations.

Coagulation factor X, activated form (FXa) was identified as the second most likely predicted target of EGCG, with its docked conformation in FXa illustrated in Fig. 4B. Wu et al. [30] showed that EGCG interacts with and inhibits FXa using a capillary electrophoresis based dual-enzyme (for thrombin and factor Xa) co-immobilized microreactor. EGCG demonstrated strong inhibitory activity against both thrombin and FXa in this high-accuracy screening assay (Z′ factor ≥ 0.94), with an inhibition rate of 96.40 ± 0.66% for FXa at a concentration of 0.5 mM, identifying it as an effective dual-target inhibitor. The optimized assay reliably distinguished compounds with selective or dual inhibitory effects on THR and FXa. Cancer cells can exploit the blood coagulation system to create a favorable microenvironment for tumor growth [39]. Therefore, the ability of EGCG to inhibit FXa may contribute to its anticancer effect by promoting anti-tumor immunity.

Collagenase (MMP1) was identified as the sixth most likely protein target for EGCG, with its docked conformation in MMP1 shown in Fig. 4C. In an experimental study conducted by Nguyen et al. [40], EGCG was identified as the most potent inhibitor of the catalytic domain of MMP1 among the eight tested flavonoids, showing 92.44% inhibition at 200 μM and an IC_50_ of 14.13 μM. Kinetic analysis using Lineweaver–Burk and Dixon plots revealed that EGCG acts as a competitive inhibitor of MMP1ca, with a calculated Ki value of 10.47 ± 0.51 μM, confirming EGCG’s strong and concentration-dependent inhibitory effect on MMP1 activity. Excessive production of MMPs is a characteristic feature of cancer cells that have the ability to form metastases [41]. Therefore, the inhibitory effect of EGCG on collagenases has the potential to reduce the metastasis rate of cancer, which is a promising avenue for therapeutic intervention.

Phospholipase A2 Group IIA (PLA2G2A) was identified as the ninth highest ranked protein target (Fig. 4D). Wang et al. [32] identified EGCG as the most potent inhibitor of porcine pancreatic phospholipase A2 (PLA2) among the catechins tested, showing a concentration-dependent inhibitory effect. The assay measured PLA2 activity in vitro using a substrate mixture, with catechin amounts ranging from 0.075 to 1.80 μmol per 300 μL, which is approximately equivalent to the total amount ingested in 0.4 to 10 cups of green tea. EGCG exhibited the lowest IC_50_ value at 0.48 μmol, followed by ECG at 0.84 μmol, while other catechins (EC, EGC, and CAT) had IC_50_ values greater than 1.8 μmol, indicating much weaker inhibition. At the highest amount tested (1.80 μmol), EGCG inhibited over 83% of PLA2 activity, demonstrating EGCG’s strong inhibitory potential against pancreatic PLA2 compared to other tea catechins. Considering the increased expression of PLA2G2A in certain cancers and its role in promoting cancer cell growth and proliferation, it is considered an important factor in oncogenesis. Therefore, given its proven inhibitory effect on this enzyme, EGCG may serve as a promising agent in cancer therapies targeting PLA2G2A.

Hemoglobin (Hb) was ranked as the 19 th most likely target protein for EGCG (Fig. 4E). Jia and Alayash [33] demonstrated that EGCG has the ability to convert ferryl-Hb (Fe^4+^) back to ferric-Hb (Fe^3+^). The effect of EGCG on human Hb was studied by monitoring its ability to inhibit Hb autoxidation and reduce ferryl-Hb. Ferryl Hb was generated by reacting ferric-Hb with hydrogen peroxide, and EGCG was added to assess its reduction kinetics using spectrophotometry and stopped-flow analysis. EGCG effectively reduced ferryl-Hb and inhibited oxidative changes in Hb, demonstrating its antioxidant potential. Both ferryl heme and its radicals are highly reactive species causing lipid, nucleic acid, and protein oxidations, and even cell and tissue damage [33]. Through its ability to convert ferryl-Hb back to its iron form, EGCG may help to mitigate oxidative stress, a process associated with DNA damage and linked to the development of cancer.

Cyclin-dependent kinase 2 (CDK2) was ranked the 20 th protein target for EGCG (Fig. 4F). Effective dose-dependent inhibition of CDK2 activity by EGCG has been demonstrated in kinase assays with histone H1 as a substrate using human A431 epidermoid carcinoma cells [34]. The cells were treated with EGCG, and lysates were prepared for immunoblot analysis and kinase activity assays. Key steps included immunoprecipitation to isolate cyclin-CDK complexes, followed by kinase assays using radioactive ATP to measure kinase activity, and detection of protein expression by chemiluminescence and autoradiography. CDK2 is a serine/threonine protein kinase that is critical for the phosphorylation of various target proteins involved in cell cycle regulation [42]. By inducing G0/G1-phase cell cycle arrest, EGCG irreversibly prevents further cell division and promotes apoptosis in human A431 epidermoid carcinoma cells [34]. This dual effect on cell cycle control and apoptosis underlines its potential as a therapeutic agent against cancer.

### Novel predicted protein targets of EGCG

Of the 20 highest ranked target proteins of EGCG, 14 have not yet been experimentally confirmed to interact with EGCG, including five kinases, all of which are involved in cancer development and where EGCG could exert its anticancer effects (see Table 1). One of these kinases is the dual mitogen-activated protein kinase kinase 6 (MAP2K6), which is part of the MAP kinase signaling pathway. MAP2K6 influences ATF2 downstream and regulates processes such as cell cycle progression, apoptosis, and tumorigenesis [43, 44]. Another is tyrosine protein kinase HCK, a member of the Src family, which is known for its role in the transformation of malignant cells after activation [45]. Phosphatidylinositol 4,5-bisphosphate 3-kinase (PIK3CG) regulates the PKB/AKT signaling pathway that is critical for growth, survival, and activation of cell proliferation [46]. The proto-oncogenic serine/threonine protein kinase PIM-1 is another kinase regulated by the JAK/STAT signaling pathway. The oncogenic function of PIM-1 includes transcriptional control of MYC, modulation of the cell cycle, and inhibition of proapoptotic factors such as BAD and FOXO3 [47]. Finally, adenosine kinase (ADK) regulates extracellular adenosine concentration and intracellular adenine nucleotide levels. Giglioni et al. [48] observed significantly increased ADK expression in cancer tissues, emphasizing their potential importance in cancer biology. These kinases represent promising targets for future experimental investigations of the interaction mechanisms of EGCG and possible therapeutic applications in cancer treatment.

Surface plasmon resonance (SPR) is a powerful technique for confirming EGCG’s predicted protein targets, particularly kinases. It allows real-time monitoring of EGCG binding to immobilized kinase targets and provides key kinetic parameters, including association/dissociation rates, equilibrium dissociation constants (Kd), and IC_50_ values, which are key for evaluating its inhibitory potential [49]. For instance, SPR has been used to demonstrate EGCG’s noncompetitive inhibition of NAD kinase [50]. Applying SPR to other kinases could offer deeper insights into EGCG’s anticancer mechanisms; however, the complexity of kinase interactions and experimental conditions may impact result accuracy [49].

### Network analysis of predicted targets

**Fig. 5:**
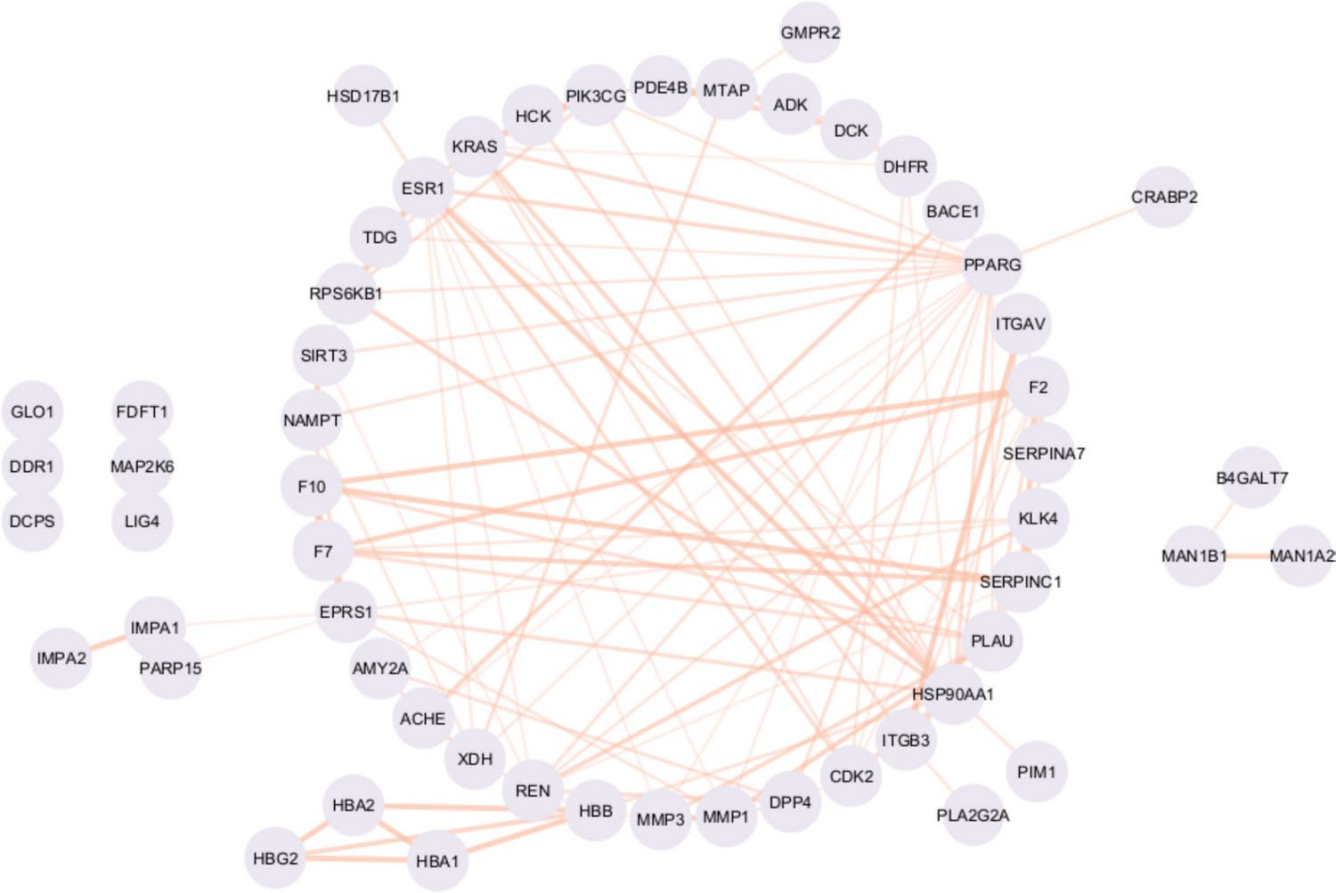
A STRING network for the top 50 predicted protein targets. The width of each edge in the network represents the STRING score, indicating the confidence level of the interactions based on available evidence

We performed a STRING [37] network analysis for the top 50 predicted protein targets to determine their connectivity and potential importance for the effects of EGCG (Fig. 5). Among the identified protein targets, PPARG has the highest number of associations with 18 connections, followed by HSP90 AA1 with 14, ESR1 with 12, and PLAU, F2, KRAS and REN9 with nine connections each (for protein targets see Table S1). On average, each protein is connected to about four others, while six proteins have no connections in the network.

### Pathway enrichment analysis of predicted targets

**Fig. 6:**
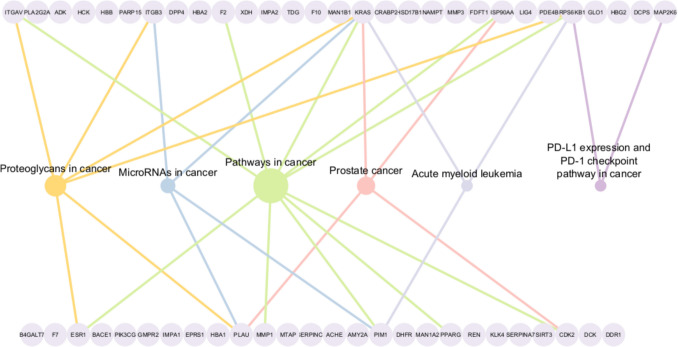
KEGG pathway enrichment with a focus on cancer-associated pathways. Each pathway is shown with a different color, and the size of each node corresponds to the number of its corresponding connections to the proteins involved in that particular pathway

The enrichment analysis of the KEGG [38] pathways of the top 50 protein targets showed enrichment in 20 pathways, with a focus on cancer-associated pathways (Fig. 6). A comprehensive table with a detailed listing of all enriched pathways can be found in Table S2. Among these pathways, *Pathways in cancer* proves to be the most enriched pathway, comprising ten associated proteins. This is followed by *Proteoglycans in cancer* with six proteins, *Prostate cancer* with five, *MicroRNAs in cancer* with four, and both *PD-L1 expression and PD-1 checkpoint signaling pathway in cancer* and *Acute myeloid leukemia* with three proteins each. Remarkably, KRAS has the most associations with five pathways, followed by RPS6 KB1 with four and both PLAU and PIM1 with three associations each. This underscores the multi-layered involvement of these genes in various cancer-related metabolic pathways and highlights potential targets for the development of broad-spectrum cancer therapies.

The mechanistic roles of KRAS, PIM1, RPS6 KB1, and PLAU in cancer biology are multifaceted, involving critical pathways that drive tumorigenesis and cancer progression. KRAS is a key regulator of the RAS/MAPK signaling pathway, driving uncontrolled proliferation, survival, and metastasis in multiple cancers, particularly pancreatic, lung, and colorectal cancers [51]. PIM1, a serine/threonine kinase, promotes tumorigenesis by enhancing cell cycle progression, inhibiting apoptosis, and modulating MYC signaling, making it a crucial player in hematological malignancies and prostate cancer [52]. RPS6 KB1, encoding the p70S6 kinase, is a major effector of mTOR signaling, promoting protein synthesis and cell growth, and is frequently amplified in breast and ovarian cancers [53]. PLAU, which encodes urokinase-type plasminogen activator, facilitates extracellular matrix degradation and enhances tumor invasion and metastasis, with high expression correlating with poor prognosis in breast, lung, and gastric cancers [54]. These oncogenes contribute to cancer progression through dysregulation of key signaling pathways, making them attractive therapeutic targets.

A possible limitation of our approach could be that the observed anticancer effects in vivo may not only be due to EGCG itself, but also due to its metabolites. EGCG is partially degraded by the gut microbiota before it is absorbed into the bloodstream [55], suggesting that these metabolites may also contribute to its therapeutic effects. To address this confounding factor, docking studies could be performed not only on EGCG but also on its known metabolites to assess their potential interactions with cancer-related proteins. This underscores the need for further research to better understand the mechanisms by which EGCG exerts its effects on multiple target proteins and to fully determine its role in cancer biology.

## Conclusions

We used an inverse molecular docking approach to identify potential human protein targets of (−)-epigallocatechin-3-gallate (EGCG). Six of the 20 most likely predicted human protein targets were experimentally confirmed to interact with EGCG, namely KRAS, FXa, MMP1, PLA2G2A, Hb, and CDK2. Fourteen protein targets have not yet been experimentally confirmed and represent potential new targets of EGCG through which it could exert its anticancer effect. These are MAP2 K6, NAMPT, IMPA1, PDE4B, HCK, DCPS, MTAP, PIK3 CG, LIG4, PIM1, MAN1B, SIRT3, ADK, and MMP3. We found that the predicted targets were enriched with cancer-related pathways. Among the identified pathways, KRAS, RPS6 KB1, PLAU, and PIM1 were most frequently annotated, indicating their possible involvement in EGCG mechanisms related to its anticancer effects. These results provide the basis for further experimental research aimed at understanding the molecular mechanisms by which EGCG interacts with its protein targets, which could potentially lead to the development of new cancer therapies.

## Supplementary information

ESM 1 (PDF 270 kb)

## Data Availability

The results of the inverse molecular docking, i.e. the complete ranked list of protein targets and docked structures, are freely accessible at http://insilab.org/files/share/egcg-data.zip.

## References

[1] Yang CS, Maliakal P, Meng X (2002) Inhibition of carcinogenesis by tea. Annu Rev Pharmacol Toxicol 42:25–54. 10.1146/annurev.pharmtox.42.082101.154309 11807163 10.1146/annurev.pharmtox.42.082101.154309

[2] Balentine DA, Wiseman SA, Bouwens LC (1997) The chemistry of tea flavonoids. Crit Rev Food Sci Nutr 37:693–704. 10.1080/10408399709527797 9447270 10.1080/10408399709527797

[3] Chen Q, Guo Z, Zhao J (2008) Identification of green tea’s (*Camellia sinensis* (L.)) quality level according to measurement of main catechins and caffeine contents by HPLC and support vector classification pattern recognition. J Pharm Biomed Anal 48:1321–1325. 10.1016/j.jpba.2008.09.016 18952392 10.1016/j.jpba.2008.09.016

[4] Šturm L, Prislan I, González-Ortega R, Mrak P, Snoj T, Anderluh G, Poklar Ulrih N (2022) Interactions of (−)-epigallocatechin-3-gallate with model lipid membranes. Biochim Biophys Acta Biomembr 1864:183999. 10.1016/j.bbamem.2022.183999 35820494 10.1016/j.bbamem.2022.183999

[5] Kurahashi N, Sasazuki S, Iwasaki M, Inoue M, Tsugane S, JPHC Study Group (2008) Green tea consumption and prostate cancer risk in Japanese men: a prospective study. Am J Epidemiol 167:71–77. 10.1093/aje/kwm249 17906295 10.1093/aje/kwm249

[6] Mhatre S, Naik S, Patravale V (2021) A molecular docking study of EGCG and theaflavin digallate with the druggable targets of SARS-CoV-2. Comput Biol Med 129:104137. 10.1016/j.compbiomed.2020.104137 33302163 10.1016/j.compbiomed.2020.104137 PMC7682485

[7] Du G-J, Zhang Z, Wen X-D, Yu C, Calway T, Yuan C-S, Wang C-Z (2012) Epigallocatechin gallate (EGCG) is the most effective cancer chemopreventive polyphenol in green tea. Nutrients 4:1679–1691. 10.3390/nu4111679 23201840 10.3390/nu4111679 PMC3509513

[8] Gan R-Y, Li H-B, Sui Z-Q, Corke H (2018) Absorption, metabolism, anti-cancer effect and molecular targets of epigallocatechin gallate (EGCG): an updated review. Crit Rev Food Sci Nutr 58:924–941. 10.1080/10408398.2016.1231168 27645804 10.1080/10408398.2016.1231168

[9] Zhou D-H, Wang X, Yang M, Shi X, Huang W, Feng Q (2013) Combination of low concentration of (-)-epigallocatechin gallate (EGCG) and curcumin strongly suppresses the growth of non-small cell lung cancer in vitro and in vivo through causing cell cycle arrest. Int J Mol Sci 14:12023–12036. 10.3390/ijms140612023 23739680 10.3390/ijms140612023 PMC3709771

[10] Basu A, Haldar S (2009) Combinatorial effect of epigallocatechin-3-gallate and TRAIL on pancreatic cancer cell death. Int J Oncol 34:281–286 19082499

[11] Tang Y, Zhao DY, Elliott S, Zhao W, Curiel TJ, Beckman BS, Burow ME (2007) Epigallocatechin-3 gallate induces growth inhibition and apoptosis in human breast cancer cells through survivin suppression. Int J Oncol 31:705–711 17786300

[12] Shankar S, Suthakar G, Srivastava RK (2007) Epigallocatechin-3-gallate inhibits cell cycle and induces apoptosis in pancreatic cancer. Front Biosci 12:5039–5051. 10.2741/2446 17569628 10.2741/2446

[13] Wj L, Jy S, Bt Z (2005) Mechanisms for the inhibition of DNA methyltransferases by tea catechins and bioflavonoids. Mol Pharmacol 68. 10.1124/mol.104.008367 10.1124/mol.104.008367 16037419

[14] Khan MA, Hussain A, Sundaram MK, Alalami U, Gunasekera D, Ramesh L, Hamza A, Quraishi U (2015) (-)-Epigallocatechin-3-gallate reverses the expression of various tumor-suppressor genes by inhibiting DNA methyltransferases and histone deacetylases in human cervical cancer cells. Oncol Rep 33:1976–1984. 10.3892/or.2015.3802 25682960 10.3892/or.2015.3802

[15] Westermarck J, Kähäri VM (1999) Regulation of matrix metalloproteinase expression in tumor invasion. FASEB J 13:781–792 10224222

[16] Ogawa K, Hara T, Shimizu M, Nagano J, Ohno T, Hoshi M, Ito H, Tsurumi H, Saito K, Seishima M, Moriwaki H (2012) (-)-Epigallocatechin gallate inhibits the expression of indoleamine 2,3-dioxygenase in human colorectal cancer cells. Oncol Lett 4:546–550. 10.3892/ol.2012.761 23741252 10.3892/ol.2012.761 PMC3673646

[17] Min NY, Kim J-H, Choi J-H, Liang W, Ko YJ, Rhee S, Bang H, Ham SW, Park AJ, Lee K-H (2012) Selective death of cancer cells by preferential induction of reactive oxygen species in response to (-)-epigallocatechin-3-gallate. Biochem Biophys Res Commun 421:91–97. 10.1016/j.bbrc.2012.03.120 22487794 10.1016/j.bbrc.2012.03.120

[18] He L, Zhang E, Shi J, Li X, Zhou K, Zhang Q, Le AD, Tang X (2013) (−)-Epigallocatechin-3-gallate inhibits human papillomavirus (HPV)-16 oncoprotein-induced angiogenesis in non-small cell lung cancer cells by targeting HIF-1α. Cancer Chemother Pharmacol 71:713–725. 10.1007/s00280-012-2063-z 23292117 10.1007/s00280-012-2063-z

[19] Harper CE, Patel BB, Wang J, Eltoum IA, Lamartiniere CA (2007) Epigallocatechin-3-Gallate suppresses early stage, but not late stage prostate cancer in TRAMP mice: mechanisms of action. Prostate 67:1576–1589. 10.1002/pros.20643 17705241 10.1002/pros.20643

[20] Jang J-Y, Lee J-K, Jeon Y-K, Kim C-W (2013) Exosome derived from epigallocatechin gallate treated breast cancer cells suppresses tumor growth by inhibiting tumor-associated macrophage infiltration and M2 polarization. BMC Cancer 13:421. 10.1186/1471-2407-13-421 24044575 10.1186/1471-2407-13-421 PMC3848851

[21] Sen T, Chatterjee A (2011) Epigallocatechin-3-gallate (EGCG) downregulates EGF-induced MMP-9 in breast cancer cells: involvement of integrin receptor α5β1 in the process. Eur J Nutr 50:465–478. 10.1007/s00394-010-0158-z 21170718 10.1007/s00394-010-0158-z

[22] Hurle MR, Yang L, Xie Q, Rajpal DK, Sanseau P, Agarwal P (2013) Computational drug repositioning: from data to therapeutics. Clin Pharmacol Ther 93:335–341. 10.1038/clpt.2013.1 23443757 10.1038/clpt.2013.1

[23] Konc J, Lešnik S, Škrlj B, Sova M, Proj M, Knez D, Gobec S, Janežič D (2022) ProBiS-Dock: a hybrid multitemplate homology flexible docking algorithm enabled by protein binding site comparison. J Chem Inf Model 62:1573–1584. 10.1021/acs.jcim.1c01176 35289616 10.1021/acs.jcim.1c01176

[24] Konc J, Lešnik S, Škrlj B, Janežič D (2021) ProBiS-Dock database: a web server and interactive web repository of small ligand–protein binding sites for drug design. J Chem Inf Model 61:4097–4107. 10.1021/acs.jcim.1c00454 34319727 10.1021/acs.jcim.1c00454

[25] Burley SK, Berman HM, Bhikadiya C, Bi C, Chen L, Di Costanzo L, Christie C, Dalenberg K, Duarte JM, Dutta S, Feng Z, Ghosh S, Goodsell DS, Green RK, Guranović V, Guzenko D, Hudson BP, Kalro T, Liang Y et al (2019) RCSB Protein Data Bank: biological macromolecular structures enabling research and education in fundamental biology, biomedicine, biotechnology and energy. Nucleic Acids Res 47:D464–D474. 10.1093/nar/gky1004 30357411 10.1093/nar/gky1004 PMC6324064

[26] Fine J, Konc J, Samudrala R, Chopra G (2020) CANDOCK: chemical atomic network-based hierarchical flexible docking algorithm using generalized statistical potentials. J Chem Inf Model 60:1509–1527. 10.1021/acs.jcim.9b00686 32069042 10.1021/acs.jcim.9b00686 PMC12034428

[27] Kores K, Lešnik S, Bren U, Janežič D, Konc J (2019) Discovery of novel potential human targets of resveratrol by inverse molecular docking. J Chem Inf Model 59:2467–2478. 10.1021/acs.jcim.8b00981 30883115 10.1021/acs.jcim.8b00981

[28] Furlan V, Konc J, Bren U (2018) Inverse molecular docking as a novel approach to study anticarcinogenic and anti-neuroinflammatory effects of curcumin. Molecules 23:3351. 10.3390/molecules23123351 30567342 10.3390/molecules23123351 PMC6321024

[29] Wang W, Xiong X, Li X, Zhang Q, Yang W, Du L (2019) In silico investigation of the anti-tumor mechanisms of epigallocatechin-3-gallate. Molecules 24:1445. 10.3390/molecules24071445 30979098 10.3390/molecules24071445 PMC6480119

[30] Wu Z-Y, Zhang H, Yang Y-Y, Yang F-Q (2020) An online dual-enzyme co-immobilized microreactor based on capillary electrophoresis for enzyme kinetics assays and screening of dual-target inhibitors against thrombin and factor Xa. J Chromatogr A 1619:460948. 10.1016/j.chroma.2020.460948 32059867 10.1016/j.chroma.2020.460948

[31] Sazuka M, Imazawa H, Shoji Y, Mita T, Hara Y, Isemura M (1997) Inhibition of collagenases from mouse lung carcinoma cells by green tea catechins and black tea theaflavins. Biosci Biotechnol Biochem 61:1504–1506. 10.1271/bbb.61.1504 9339552 10.1271/bbb.61.1504

[32] Wang S, Noh SK, Koo SI (2006) Green tea catechins inhibit pancreatic phospholipase A(2) and intestinal absorption of lipids in ovariectomized rats. J Nutr Biochem 17:492–498. 10.1016/j.jnutbio.2006.03.004 16713229 10.1016/j.jnutbio.2006.03.004

[33] Jia Y, Alayash AI (2008) Effects of (-)-epigallocatechin gallate on the redox reactions of human hemoglobin. Free Radic Biol Med 45:659–666. 10.1016/j.freeradbiomed.2008.05.010 18539156 10.1016/j.freeradbiomed.2008.05.010

[34] Ahmad N, Cheng P, Mukhtar H (2000) Cell cycle dysregulation by green tea polyphenol epigallocatechin-3-gallate. Biochem Biophys Res Commun 275:328–334. 10.1006/bbrc.2000.3297 10964666 10.1006/bbrc.2000.3297

[35] Shannon P, Markiel A, Ozier O, Baliga NS, Wang JT, Ramage D, Amin N, Schwikowski B, Ideker T (2003) Cytoscape: a software environment for integrated models of biomolecular interaction networks. Genome Res 13:2498–2504. 10.1101/gr.1239303 14597658 10.1101/gr.1239303 PMC403769

[36] Doncheva NT, Morris JH, Gorodkin J, Jensen LJ (2019) Cytoscape StringApp: network analysis and visualization of proteomics data. J Proteome Res 18:623–632. 10.1021/acs.jproteome.8b00702 30450911 10.1021/acs.jproteome.8b00702 PMC6800166

[37] Franceschini A, Szklarczyk D, Frankild S, Kuhn M, Simonovic M, Roth A, Lin J, Minguez P, Bork P, von Mering C, Jensen LJ (2013) STRING v9.1: protein-protein interaction networks, with increased coverage and integration. Nucleic Acids Res 41:D808–D815. 10.1093/nar/gks1094 23203871 10.1093/nar/gks1094 PMC3531103

[38] Kanehisa M, Goto S (2000) KEGG: Kyoto Encyclopedia of Genes and Genomes. Nucleic Acids Res 28:27–30. 10.1093/nar/28.1.27 10592173 10.1093/nar/28.1.27 PMC102409

[39] Graf C, Wilgenbus P, Pagel S, Pott J, Marini F, Reyda S, Kitano M, Macher-Göppinger S, Weiler H, Ruf W (2019) Myeloid cell-synthesized coagulation factor X dampens anti-tumor immunity. Sci Immunol 4:eaaw8405. 10.1126/sciimmunol.aaw8405 31541031 10.1126/sciimmunol.aaw8405 PMC6830514

[40] Nguyen TTH, Moon Y-H, Ryu Y-B, Kim Y-M, Nam S-H, Kim M-S, Kimura A, Kim D (2013) The influence of flavonoid compounds on the *in vitro* inhibition study of a human fibroblast collagenase catalytic domain expressed in *E. coli*. Enzym Microb Technol 52:26–31. 10.1016/j.enzmictec.2012.10.001 10.1016/j.enzmictec.2012.10.001 23199735

[41] Zucker S, Lysik RM, Zarrabi MH, Moll U (1993) M(r) 92,000 type IV collagenase is increased in plasma of patients with colon cancer and breast cancer. Cancer Res 53:140–146 8416738

[42] Okuda M, Horn HF, Tarapore P, Tokuyama Y, Smulian AG, Chan PK, Knudsen ES, Hofmann IA, Snyder JD, Bove KE, Fukasawa K (2000) Nucleophosmin/B23 is a target of CDK2/cyclin E in centrosome duplication. Cell 103:127–140. 10.1016/s0092-8674(00)00093-3 11051553 10.1016/s0092-8674(00)00093-3

[43] Raingeaud J, Whitmarsh AJ, Barrett T, Dérijard B, Davis RJ (1996) MKK3- and MKK6-regulated gene expression is mediated by the p38 mitogen-activated protein kinase signal transduction pathway. Mol Cell Biol 16:1247–1255. 10.1128/MCB.16.3.1247 8622669 10.1128/mcb.16.3.1247 PMC231107

[44] Bhoumik A, Bergami PL, Ronai Z (2007) ATF2 on the double – activating transcription factor and DNA damage response protein. Pigment Cell Res 20:498–506. 10.1111/j.1600-0749.2007.00414.x 17935492 10.1111/j.1600-0749.2007.00414.x PMC2997391

[45] Summy JM, Gallick GE (2003) Src family kinases in tumor progression and metastasis. Cancer Metastasis Rev 22:337–358. 10.1023/a:1023772912750 12884910 10.1023/a:1023772912750

[46] Bondeva T, Pirola L, Bulgarelli-Leva G, Rubio I, Wetzker R, Wymann MP (1998) Bifurcation of lipid and protein kinase signals of PI3Kgamma to the protein kinases PKB and MAPK. Science 282:293–296. 10.1126/science.282.5387.293 9765155 10.1126/science.282.5387.293

[47] Wang Z, Bhattacharya N, Mixter PF, Wei W, Sedivy J, Magnuson NS (2002) Phosphorylation of the cell cycle inhibitor p21Cip1/WAF1 by Pim-1 kinase. Biochim Biophys Acta 1593:45–55. 10.1016/s0167-4889(02)00347-6 12431783 10.1016/s0167-4889(02)00347-6

[48] Giglioni S, Leoncini R, Aceto E, Chessa A, Civitelli S, Bernini A, Tanzini G, Carraro F, Pucci A, Vannoni D (2008) Adenosine kinase gene expression in human colorectal cancer. Nucleosides Nucleotides Nucleic Acids 27:750–754. 10.1080/15257770802145629 18600536 10.1080/15257770802145629

[49] Kitagawa D, Gouda M, Kirii Y (2014) Quick evaluation of kinase inhibitors by surface plasmon resonance using single-site specifically biotinylated kinases. J Biomol Screen 19:453–461. 10.1177/1087057113506051 24080257 10.1177/1087057113506051

[50] Liu T, Shi W, Ding Y, Wu Q, Zhang B, Zhang N, Wang M, Du D, Zhang H, Han B, Guo D, Zheng J, Li Q, Luo C (2022) (−)-Epigallocatechin gallate is a noncompetitive inhibitor of NAD kinase. ACS Med Chem Lett 13:1699–1706. 10.1021/acsmedchemlett.2c00163 36385933 10.1021/acsmedchemlett.2c00163 PMC9661698

[51] Prior IA, Hood FE, Hartley JL (2020) The frequency of Ras mutations in cancer. Cancer Res 80:2969–2974. 10.1158/0008-5472.CAN-19-3682 32209560 10.1158/0008-5472.CAN-19-3682 PMC7367715

[52] Nawijn MC, Alendar A, Berns A (2011) For better or for worse: the role of Pim oncogenes in tumorigenesis. Nat Rev Cancer 11:23–34. 10.1038/nrc2986 21150935 10.1038/nrc2986

[53] Biever A, Valjent E, Puighermanal E (2015) Ribosomal protein S6 phosphorylation in the nervous system: from regulation to function. Front Mol Neurosci 8:75. 10.3389/fnmol.2015.00075 26733799 10.3389/fnmol.2015.00075 PMC4679984

[54] Andreasen PA, Kjøller L, Christensen L, Duffy MJ (1997) The urokinase-type plasminogen activator system in cancer metastasis: a review. Int J Cancer 72:1–22. 10.1002/(SICI)1097-0215(19970703)72:1<1::AID-IJC1>3.0.CO;2-Z 9212216 10.1002/(sici)1097-0215(19970703)72:1<1::aid-ijc1>3.0.co;2-z

[55] Lambert JD, Sang S, Hong J, Yang CS (2010) Anticancer and anti-inflammatory effects of cysteine metabolites of the green tea polyphenol, (-)-epigallocatechin-3-gallate. J Agric Food Chem 58:10016–10019. 10.1021/jf102311t 20718469 10.1021/jf102311t PMC3045820

