# Supplementary material for: Identification of potential human targets for epigallocatechin gallate through a novel protein binding site screening approach
Source: J Mol Model. 2025 Jun 13;31(7):189. doi: 10.1007/s00894-025-06410-y (PMC12165993; doi:10.1007/s00894-025-06410-y)
Supplement: Supplementary file 1 — (PDF 270 kb) [file 894_2025_6410_MOESM1_ESM.pdf]

Supporting Information

Identification of potential human targets for epigallocatechin gallate through a novel protein binding site screening approach

Jernej Hirci, Sandra Škufca, Tanja Kunej, Dušanka Janežič, Janez Konc

Table S1. Top 95 target protein complex structures with the lowest ProBiS-Score for EGCG, predicted by the ProBiS-Dock program.

#	PDB ID, chain	Protein name[a]	Gene symbol[b]	ProBiS-Score (Konc *et. al*., 2022)
1.	4LV6, B	GTPase KRas	KRAS	-90.530
2.	2D1J, A	Coagulation factor X, heavy chain	F10	-83.500
3.	3ENM, AC	Dual specificity mitogen-activated protein kinase kinase 6	MAP2K6	-82.459
4.	4LVF, AB	Nicotinamide phosphoribosyltransferase	NAMPT	-82.124
5.	1IME, AB	Inositol monophosphatase	IMPA1	-81.353
6.	3AYK, A	Collagenase	MMP1	-81.231
7.	3WD9, A	CAMP-specific 3',5'-cyclic phosphodiesterase 4B	PDE4B	-79.799
8.	2C0T, A	Tyrosine-protein kinase HCK	HCK	-77.626
9.	1J1A, AB	Phospholipase A2	PLA2G2A	-74.696
10.	1ST4, AB	mRNA decapping enzyme	DCPS	-72.006
11.	1CB0, A	5'-deoxy-5'-methylthioadenosine phosphorylase	MTAP	-71.748
12.	4J6I, A	Phosphatidylinositol-4,5-bisphosphate 3-kinase catalytic subunit gamma isoform	PIK3CG	-71.132
13.	3W1B, A	DNA ligase 4	LIG4	-70.995
14.	3R01, A	Proto-oncogene serine/threonine-protein kinase pim-1	PIM1	-70.887
15.	1FO3, A	Alpha 1,2-mannosidase	MAN1B	-70.779
16.	4C7B, AB	NAD-dependent protein deacetylase sirtuin-3, mitochondrial	SIRT3	-70.313
17.	2I6A, A	Adenosine kinase	ADK	-70.043
18.	2JT5, A	Stromelysin-1	MMP3	-69.280
19.	4MQK, ABEF	Hemoglobin subunit alpha, subunit gamma-2	AE: *HBA1, HBA2*<br>BF: *HBB, HBG2*	-69.226
20.	3R8M, A	Cyclin-dependent kinase 2	CDK2	-69.161
21.	2ONC, A	Dipeptidyl peptidase 4	DPP4	-68.141
22.	4IRQ, A	Beta-1, 4-galactosyltransferase 7	B4GALT7	-68.052
23.	2CKJ, A	Xanthine oxidoreductase	XDH	-67.874

24	1TB6, HI	H: Thrombin<br>I: Antithrombin III	H: *F2*<br>I: *SERPINC1*	-67.819
25.	2XFI, A	Beta-secretase 1	BACE1	-67.273
26.	2CEO, A	Thyroxine-binding globulin	SERPINA7	-67.270
27.	2A7R, BC	GMP reductase 2	GMPR2	-67.269
28.	3OS8, A	Estrogen receptor	ESR1	-67.254
29.	1OWH, A	Urukinase-type plasminogen activator	PLAU	-66.906
30.	4HVC, B	Bifunctional glutamate/proline--tRNA ligase	EPRS1	-66.778
31.	3HB4, X	Estradiol 17-beta-dehidrogenase 1	HSD17B1	-66.501
32.	2DDK, AB	Inositol monophosphatase 2	IMPA2	-66.303
33.	2A2Q, H	Coagulation factor VII	F7	-66.174
34.	3FS6, A	Dihydrofolate reductase	DHFR	-66.137
35.	4GJD, A	Renin	REN	-66.061
36.	1X9D, A	Endoplasmic reticulum manosyl-oligosacharide 1,2-alpha-mannosidase	MAN1B1	-65.932
37.	3QTF, A	Heat shock protein HSP-90-alpha	HSP90AA1	-65.834
38.	3BC5, A	Peroxisome proliferator-activated receptor gamma	PPARG	-65.803
39.	3ZOS, A	Epithelial discoidin domain-containing receptor 1	DDR1	-65.402
40.	4G1M, AB	A: Integrin alpha-V<br>B: Integrin beta-3	A: *ITGAV*<br>B: *ITGB3*	-65.370
41.	4I9R, A	Cellular retinoic acid–binding protein 2	CRABP2	-65.331
42.	4EY5, B	Acetylcholinesterase	ACHE	-65.293
43.	2BDG, A	Kallikrein-4	KLK4	-65.279
44.	1QIN, AB	Lactoylglutathione lyase	GLO1	-65.201
45.	1KGU, A	Alpha-amylase, pancreatic	AMY2A	-65.063
46.	2D07, A	G/T mismatch-specific thymine DNA glycosylase	TDG	-64.890
47.	3LEE, A	Squalene synthase	FDFT1	-64.882
48.	2ZI5,A	Deoxycytidine kinase	DCK	-64.840
49.	3WF7, A	Ribosomal protein S6 kinase beta-1	RPS6KB1	-64.805
50.	3GEY, A	Protein mono-ADP-ribosyltransferase	PARP15	-64.804
51.	2RCW, A	Poly [ADP-ribose] polymerase 1	PARP1	-64.790
52.	1RC0, A	Phosphoribosylglycinamide formyltransferase	GART	-64.743
53.	2GQG, A	Proto-oncogene tyrosine–protein kinase ABL1	ABL1	-64.608
54.	2PQF, A	Protein mono-ADP-ribosyltransferase	PARP12	-64.537
55.	1O6U, A	SEC14–like protein 2	SEC14L2	-64.450
56.	1MQB, A	Ephrin type-A receptor 2	EPHA2	-64.418
57.	2ITX, A	Epidermal growth factor receptor	EGFR	-64.403
58.	2DSB, AB	ADP-sugar pyrophosphatase	NUDT5	-64.301
59.	3Q4T, A	Activin receptor type-2A	ACVR2A	-64.233
60.	1RCT, E	Purine nucleoside phosphorylase	PNP	-64.222
61.	3LC3, A	Coagulation factor IX	F9	-64.198
62.	1PY5, A	TGF-beta receptor type 1	TGFBR1	-64.118
63.	1JCN, A	Inosine monophosphate dehydrogenase 1	IMPDH1	-64.065
64.	2H96, AF	A:Mitogen–activated protein kinase 8<br>F:C-jun-amino-terminal kinase-interacting protein 1	A: *MAPK8*<br>F: *MAPK8IP1*	-63.810
65.	2FIE, DH	Fructose-1,6-biphosphatase 1	FBP1	-63.806
66.	2P55, A	Dual specificity mitogen–activated protein kinase kinase 1	MAP2K1	-63.801
67.	4EKI, A	Histone-lysine N-methyltransferase, H3 lysine-79 specific	DOT1L	-63.767
68.	3KYT, AC	A: Nuclear receptor ROR-gamma<br>C: Nuclear receptor coactivator 2	A: *RORC*<br>C: *NCOA2*	-63.750
69.	2J6L, AB	Aldehyde dehydrogenase family 7 member A1	ALDH7A1	-63.730
70.	4E5W, A	Tyrosine-protein kinase JAK1	JAK1	-63.581
71.	3HN3, A	Beta–glucuronidase	GUSB	-63.407
72.	2RJ6, A	Glycoprotein-fucosylgalactoside alpha-galactosyltransferase	ABO	-63.262

73.	3S4J, A	Farnesyl pyrophosphate synthase	FDPS	-63.140
74.	3KRL, A	Fibroblast growth factor receptor 1	A: *CSF1R, FGFR1*	-62.992
75.	2JBH, A	Phosphoribosyltransferase domain–containing protein 1	PRTFDC1	-62.890
76.	3LQ8, A	Hepatocyte growth factor receptor	MET	-62.845
77.	1BYG, A	C-terminal SRC kinase	CSK	-62.699
78.	2O3Q, A	ADP-ribosyl cyclase 1	CD38	-62.644
79.	4G1M, AB	A: Integrin alpha–V<br>B: Integrin beta–3	A: *ITGAV*<br>B: *ITGB3*	-62.526
80.	4D4M, A	6-phosphofructo-2-kinase/fructose-2,6-biphosphatase 3	PFKFB3	-62.447
81.	1FLS, A	Collagenase-3	MMP13	-62.382
82.	4J03, A	Bifunctional epoxide hydrolase 2	EPHX2	-62.282
83.	2CBS, A	Cellular retinoic acid-binding protein 2	CRABP2	-62.208
84.	3CD5, AB	3-hydroxy-3-methylglutaryl-coenzyme A reductase	HMGCR	-62.200
85.	3RZL, A	DNA oxidative demethylase	ALKBH2	-62.092
86.	1Q4N, X	Alpha-amylase 1A	AMY1A	-62.020
87.	2QLR, CD	Kynurenine/alpha-aminoadipate aminotransferase mitochondrial	AADAT	-62.016
88.	1E7H, A	Serum albumin	ALB	-61.992
89.	3IU2, A	Glycylpeptide N-tetradecanoyltransferase 1	NMT1	-61.859
90.	3PDJ, AB	Corticosteroid 11-beta-dehydrogenase isozyme 1	HSD11	-61.844
91.	3KFJ, A	Growth factor receptor-bound protein 2	GRB2	-61.818
92.	3HII, AB	Diamine oxidase	AOC1	-61.789
93.	4MCS, A	Glutamate carboxypeptidase 2	FOLH1	-61.761
94.	2D7R, A	Polypeptide N-acetylgalactosaminyltransferase 10	GALNT10	-61.730
95.	3DCT, A	Bile acid receptor	NR1H4	-61.653

[a]Gene names were obtained from PDB (https://www.rcsb.org/).
[b]Gene symbols were obtained from Uniprot (https://www.uniprot.org/).

Table S2. Enriched KEGG pathways for the proteins (55) in the top 50 predicted protein complex structures.

KEGG Pathways Description	Number of genes	Genes	FDR value	*p*-value
Metabolic pathways	20	B4GALT7, NAMPT, HSD17B1, IMPA2, DCK, PDE4B, MAN1A2, EPRS1, MAN1B1, GLO1, XDH, SIRT3, PLA2G2A, DHFR, IMPA1, PIK3CG, ADK, AMY2A, FDFT1, MTAP	2.71E-07	8.06E-10
Pathways in cancer	10	RPS6KB1, KRAS, ITGAV, CDK2, PPARG, F2, MMP1, HSP90AA1, PIM1, ESR1	2.70E-04	1.61E-06
Complement and coagulation cascades	5	F2, SERPINC1, PLAU, F10, F7	4.80E-04	4.32E-06
Prostate cancer	5	KRAS, CDK2, MMP3, HSP90AA1, PLAU	7.90E-04	9.42E-06
Proteoglycans in cancer	6	RPS6KB1, KRAS, ITGAV, PLAU, ESR1, ITGB3	0.0013	1.92E-05
PI3K-Akt signaling pathway	7	RPS6KB1, KRAS, ITGAV, CDK2, HSP90AA1, PIK3CG, ITGB3	0.0031	5.62E-05
Purine metabolism	4	DCK, PDE4B, XDH, ADK	0.0181	4.80E-04
Thyroid hormone signaling pathway	4	KRAS, ITGAV, ESR1, ITGB3	0.0181	3.90E-04
Human cytomegalovirus	5	RPS6KB1, KRAS, ITGAV, ITGB3, MAP2K6	0.0181	3.80E-04

infection				
Fluid shear stress and atherosclerosis	4	ITGAV, HSP90AA1, ITGB3, MAP2K6	0.0181	5.10E-04
Acute myeloid leukemia	3	RPS6KB1, KRAS, PIM1	0.0299	9.80E-04
MicroRNAs in cancer	4	KRAS, PLAU, PIM1, ITGB3	0.0308	0.0011
Inositol phosphate metabolism	3	IMPA2, IMPA1, PIK3CG	0.0309	0.0012
Endocrine resistance	3	RPS6KB1, KRAS, ESR1	0.0472	0.0025
Longevity regulating pathway	3	RPS6KB1, KRAS, PPARG	0.0472	0.002
IL-17 signaling pathway	3	MMP3, MMP1, HSP90AA1	0.0472	0.0023
Regulation of actin cytoskeleton	4	KRAS, ITGAV, F2, ITGB3	0.0472	0.0029
Progesterone-mediated oocyte maturation	3	KRAS, CDK2, HSP90AA1	0.0472	0.0026
Human papillomavirus infection	5	RPS6KB1, KRAS, ITGAV, CDK2, ITGB3	0.0472	0.0022
Kaposi sarcoma-associated herpesvirus infection	4	KRAS, HCK, PIK3CG, MAP2K6	0.0472	0.002
PD-L1 expression and PD-1 checkpoint pathway in cancer	3	RPS6KB1, KRAS, MAP2K6	0.0472	0.002
